# Depression Reduces Accuracy While Parkinsonism Slows Response Time for Processing Positive Feedback in Patients with Parkinson’s Disease with Comorbid Major Depressive Disorder Tested on a Probabilistic Category-Learning Task

**DOI:** 10.3389/fpsyt.2017.00084

**Published:** 2017-06-12

**Authors:** Mohammad M. Herzallah, Hussain Y. Khdour, Ahmad B. Taha, Amjad M. Elmashala, Hamza N. Mousa, Mohamad B. Taha, Zaid Ghanim, Mahmud M. Sehwail, Adel J. Misk, Tarryn Balsdon, Ahmed A. Moustafa, Catherine E. Myers, Mark A. Gluck

**Affiliations:** ^1^Palestinian Neuroscience Initiative, Al-Quds University, Abu Dis, Palestine; ^2^Center for Molecular and Behavioral Neuroscience, Rutgers University, Newark, NJ, United States; ^3^School of Social Sciences and Psychology, Marcs Institute for Brain and Behavior, Western Sydney University, Sydney, NSW, Australia; ^4^Department of Veterans Affairs, VA New Jersey Health Care System, East Orange, NJ, United States; ^5^Department of Physiology, Pharmacology and Neuroscience, New Jersey Medical School, Rutgers University, Newark, NJ, United States

**Keywords:** Parkinson’s disease, depression, positive feedback, negative feedback, category learning, dopamine

## Abstract

Major depressive disorder (MDD) is the most common non-motor manifestation of Parkinson’s disease (PD) affecting 50% of patients. However, little is known about the cognitive correlates of MDD in PD. Using a computer-based cognitive task that dissociates learning from positive and negative feedback, we tested four groups of subjects: (1) patients with PD with comorbid MDD, (2) patients with PD without comorbid MDD, (3) matched patients with MDD alone (without PD), and (4) matched healthy control subjects. Furthermore, we used a mathematical model of decision-making to fit both choice and response time data, allowing us to detect and characterize differences between the groups that are not revealed by cognitive results. The groups did not differ in learning accuracy from negative feedback, but the MDD groups (PD patients with MDD and patients with MDD alone) exhibited a selective impairment in learning accuracy from positive feedback when compared to the non-MDD groups (PD patients without MDD and healthy subjects). However, response time in positive feedback trials in the PD groups (both with and without MDD) was significantly slower than the non-PD groups (MDD and healthy groups). While faster response time usually correlates with poor learning accuracy, it was paradoxical in PD groups, with PD patients with MDD having impaired learning accuracy and PD patients without MDD having intact learning accuracy. Mathematical modeling showed that both MDD groups (PD with MDD and MDD alone) were significantly slower than non-MDD groups in the rate of accumulation of information for stimuli trained by positive feedback, which can lead to lower response accuracy. Conversely, modeling revealed that both PD groups (PD with MDD and PD alone) required more evidence than other groups to make responses, thus leading to slower response times. These results suggest that PD patients with MDD exhibit cognitive profiles with mixed traits characteristic of both MDD and PD, furthering our understanding of both PD and MDD and their often-complex comorbidity. To the best of our knowledge, this is the first study to examine feedback-based learning in PD with MDD while controlling for the effects of PD and MDD.

## Introduction

Patients with Parkinson’s disease (PD) suffer from a variety of non-motor symptoms, such as sleep disturbances, autonomic dysfunction, gastrointestinal, urogenital and psychiatric problems, as well as cognitive decline (1, 2). Comorbid major depressive disorder (MDD) is the most frequently reported non-motor manifestation of PD in ~40–60% of patients (3–5). Among patients with PD, comorbid MDD (PD-MDD) has the strongest association with declines in health-related quality of life (6–8) and cognitive function (9–12). Several cognitive domains are affected by PD-MDD such as episodic and working memory, language, visuospatial abilities, abstract reasoning, executive function, and reinforcement learning (13–16).

Recently, the focus of cognitive research in PD has been on reinforcement learning (17). A number of studies have shown that patients with PD exhibit deficits in associative learning, such as category-learning tasks where subjects learn through trial and error to make specific responses based on corrective feedback (18–21). Previous research has shown that this learning impairment in PD specifically reflects deficits in learning from positive feedback, but with spared learning from negative feedback (22). We have shown similar selective deficits in learning from positive feedback in patients with MDD (23).

Cognitive impairment in PD and MDD patients is not limited to cognitive accuracy, but extends to “cognitive slowing” (24) where the speed of cognitive processing and responding is affected (25, 26). The level of cognitive slowing in PD has been shown to correlate with the level of motor slowing (27, 28). In other words, motor slowing in PD is inseparable from cognitive slowing as they occur in parallel (29). On the other hand, MDD does not entail a global deficit of the sensorimotor processing. Instead, stimulus preprocessing is unaffected by MDD, while response selection is impaired (30). However, it is unclear whether cognitive deficits in PD-MDD are inherited from PD, MDD, or both.

In this study, we investigate both learning accuracy and response time (RT) in patients with PD-MDD, patients with PD without MDD, patients with MDD without PD, and matched healthy controls (HCs). We used a new version of a computer-based cognitive task that dissociates learning from positive and negative feedback and has been well-validated in both patients with PD and those with MDD (22, 23). We predicted that the learning accuracy of patients with PD-MDD will be similar to that of patients with MDD, while their RT will be similar to patients with PD. The underlying cognitive mechanisms behind these potential differences were further investigated using a mathematical diffusion model that describes the decision-making process as the accumulation of noisy evidence toward a decision threshold (31). To the best of our knowledge, this is the first study to investigate both learning accuracy and RT in patients with PD-MDD, using both experimental and mathematical modeling methods.

## Materials and Methods

### Participants

We recruited 57 eligible Arabic-speaking participants from different neurological and psychiatric clinics in the West Bank, Palestine. The participant groups were (1) PD-MDD (*n* = 13), (2) PD without MDD (*n* = 17), (3) medication-naïve MDD (*n* = 12), and (4) matched HCs (*n* = 15).

The stages of PD, as assessed by the Hoehn and Yahr (H&Y) scale (32) ranged from 1.0 to 3.5 (M = 2.18; SD = 0.73). Motor symptoms were assessed using the Unified Parkinson Disease Rating Scale [UPDRS (33)] and ranged from 7 to 67 (M = 28.6; SD = 14.6). Duration since initial diagnosis with PD or MDD ranged from 0 to 15 years (M = 4.73; SD = 3.88). All PD patients were on dopaminergic medications at the time of testing. Out of the 30 PD patients, 20 were on l-DOPA/carbidopa only (9 with MDD and 11 without MDD), 3 on l-DOPA/carbidopa plus the dopamine agonist ropinirole (1 with MDD and 3 without MDD), 3 on l-DOPA/carbidopa plus the dopamine agonist pramipexole (1 with MDD and 2 without MDD), and 3 on ropinirole alone (2 with MDD and 1 without MDD). All participants underwent clinical diagnostic *DSM-IV-TR* unstructured clinical interviews as well as structured clinical interviews using the Mini International Neuropsychiatric Interview (34) to confirm the diagnosis of MDD in the PD-MDD and the MDD groups, and the absence of MDD and other psychiatric diseases in the PD and HC groups.

Participants’ age ranged from 26 to 81 years. Participants were group matched for age (M = 54.4; SD = 4.5), gender (33 males and 24 females), and years of education (M = 12.8; SD = 2.2), as shown in Table 1. Inclusion criteria for HCs included absence of any psychiatric, neurological, or other disorders that might affect cognition. Exclusion criteria for all participants included psychotropic drug exposure; major medical or neurological illness other than PD and/or MDD; illicit drug use or alcohol abuse within the past year; lifetime history of alcohol or drug dependence; psychiatric disorders other than MDD; current pregnancy; or breastfeeding. This study was carried out in accordance with the recommendations of the Al-Quds University Research Ethics Committee with written informed consent from all subjects. All subjects gave written informed consent in accordance with the Declaration of Helsinki. The protocol was approved by the Al-Quds University Research Ethics Committee.

**Table 1:** **Neuropsychological and demographic characteristics of the participants**.

	Parkinson’s disease (PD)-major depressive disorder (MDD)	PD	MDD	Healthy control
Total number (male/female)	13 (4/9)	17 (15/2)	12 (4/8)	15 (10/5)
Age (years)	55.2 (11.9)	59.4 (12.6)	48.5 (5.1)	54.3 (12.3)
Education (years)	12.2 (3.4)	14.4 (4.6)	9.8 (3.5)	14.6 (2.7)
Time since diagnosis (years)	4.84 (3.7)	5.24 (4.0)	0	N.A.
MMSE	27.5 (1.8)	28.5 (1.1)	27.7 (1.4)	29.3 (0.7)
BDI-II	26.9 (7.7)	8.3 (5.2)	29.1 (8.7)	6.7 (5.3)
BAI	22.9 (13.7)	13.1 (8.9)	24.5 (8.2)	4.1 (3.1)
Wechsler Adult Intelligence Scale—Revised digit span	10.1 (1.8)	12.4 (2.4)	9.9 (2.1)	12.1 (2.6)

### Neuropsychological Test Battery

All participants completed the Arabic version (35) of neuropsychological tests: the Mini Mental State Examination (36), the Beck Depression Inventory II (37), the Beck Anxiety Inventory (38), and the Wechsler Adult Intelligence Scale—Revised (WAIS-R) digit span test (39).

There was a significant difference between groups in MMSE scores (Kruskal–Wallis *H* = 15.117, df = 3, *p* = 0.002). *Post hoc* Mann–Whitney tests, with Bonferroni corrected α = 0.008, revealed significant differences between the HCs and both MDD and PD-MDD groups (*p* = 0.001 and 0.002, respectively), but no significant differences between the three disease groups (MDD, PD-MDD, and PD; *p* > 0.05). As expected, BDI-II scores differed significantly across groups [*F*(3, 56) = 43.885, *p* < 0.001, η^2^ = 0.71]; specifically, Tukey’s *HSD post hoc* test revealed significant differences between HC vs. MDD and PD-MDD groups, and PD vs. MDD and PD-MDD groups (all *p* < 0.001), but no significant differences between HCs and PD or between the MDD and PD-MDD (all *p* > 0.05). In addition, BAI differed significantly across groups [*F*(3, 56) = 15.015, *p* < 0.001, η^2^ = 0.46]. Tukey’s HSD on BAI results revealed significant differences between HC and all disease groups (all *p* < 0.05), PD with both MDD and PD-MDD (all *p* < 0.05) and no significant difference between MDD and PD-MDD (*p* > 0.05). We used one-way ANOVA to compare WAIS-R digit span results among groups, which revealed a significant effect of group [*F*(3, 55) = 4.548, *p* = 0.007, η^2^ = 0.21]. Tukey’s *HSD* revealed significant differences only between the PD and MDD groups (*p* < 0.05). Independent-samples *t*-test to compare UPDRS scores showed a significant difference between the PD-MDD and PD groups [*t*(28) = 2.696, *p* = 0.012, *r*^2^ = 0.21]. Finally, Mann–Whitney test on H&Y scores revealed a significant difference between the PD-MDD and PD groups (Mann–Whitney *U* = 62.5, *p* = 0.031, *r*^2^ = 0.15). The PD-MDD group had significantly higher scores than the PD group in both UPDRS and H&Y.

### Computer-Based Cognitive Task

#### Learning from Positive and Negative Feedback

**Figure 1:**
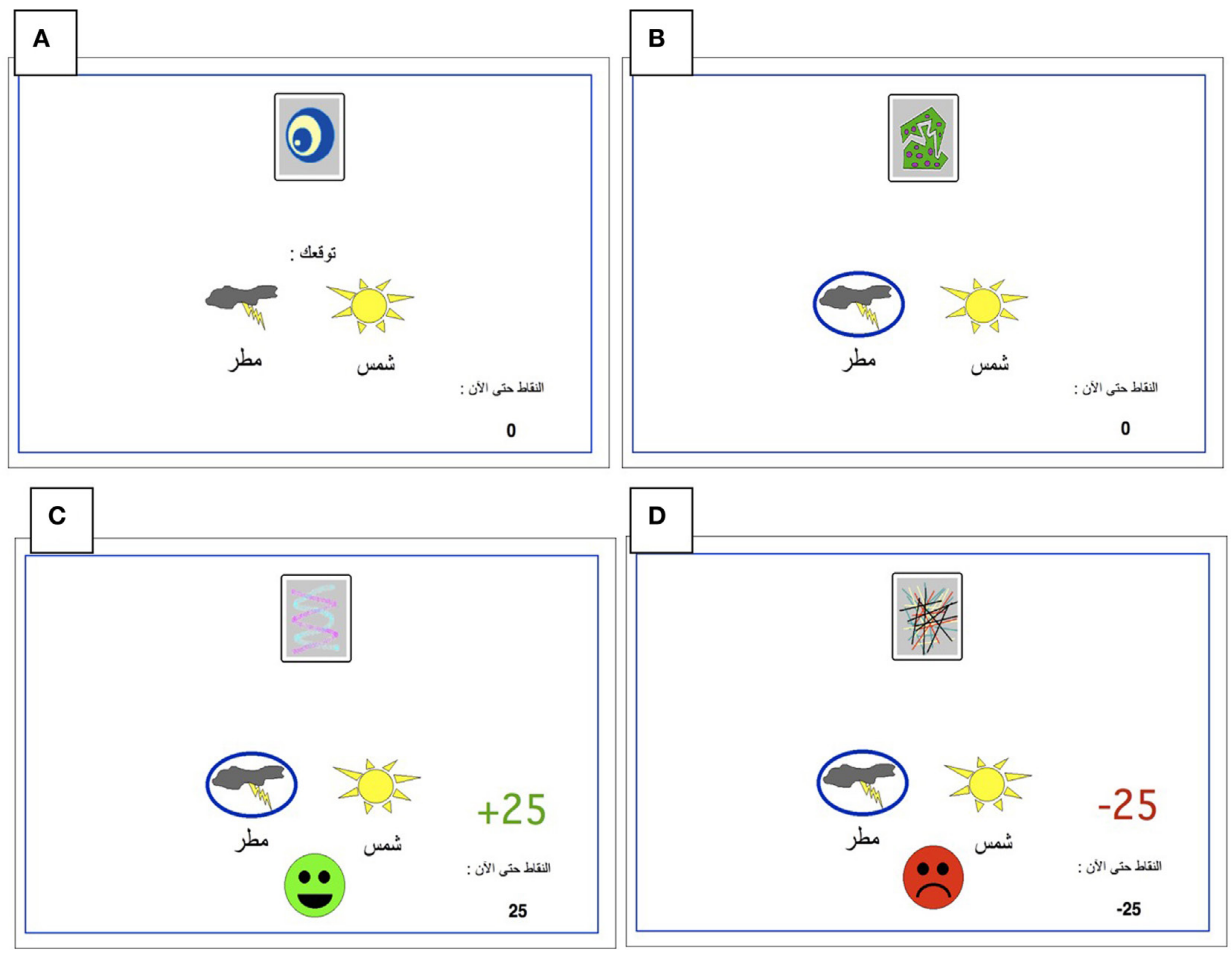
**The Arabic-translated feedback-based probabilistic category task**. **(A)** On each trial, the participant sees one of the four stimuli and is asked whether this stimulus predicts rain or sun. **(B)** No feedback is given for incorrect answers in positive feedback stimuli or correct answers in negative feedback stimuli. **(C)** For positive feedback stimuli, correct responses get rewarded with visual feedback and 25-point winnings. **(D)** For negative feedback stimuli, incorrect responses get punished with visual feedback and the loss of 25 points.

All participants were administered a new and improved version of the computer-based probabilistic classification task that was used by Bódi et al. (22). For each participant, four images were randomly assigned to be S1, S2, S3, and S4. On each trial, participants viewed one of the four stimuli (Figure 1) and were asked to guess whether it belonged to category A (rain) or B (sun). On any given trial, stimuli S1 and S3 belonged to category A with 90% probability and to category B with 10% probability, while stimuli S2 and S4 belonged to category B with 90% probability and to category A with 10% probability (Table 2). Stimuli S1 and S2 were used in positive-feedback learning trials. Thus, if the participant correctly guessed category membership on a trial with either of these stimuli, a positive feedback of +25 points was received; if the participant guessed incorrectly, no feedback appeared and there was no point gain. Stimuli S3 and S4 were used in the negative-feedback learning trials. Thus, if the participant guessed incorrectly on a trial with either of these stimuli, a negative feedback of −25 points was received; correct guesses received no feedback or point loss. The no-feedback outcome, when it arrived, was ambiguous, as it could signal lack of positive feedback (if received during a trial with S1 or S2) or lack of negative feedback (if received during a trial with S3 or S4).

**Table 2:** **Category and feedback structure of probabilistic classification task**.

Stimulus	Probability category A (%)	Probability category B (%)	Feedback
S1	90	10	If correct: +25
S2	10	90	If incorrect: no feedback
S3	90	10	If correct: no feedback
S4	10	90	If incorrect: −25

The task was conducted on a Macintosh Macbook, programmed in the SuperCard language. The participant was seated in a quiet testing room at a comfortable viewing distance from the screen. The keyboard was masked except for two keys, labeled “Sun” and “Rain” that the participant could use to enter responses. Participants first completed a practice phase that walked the participant through an example of a correct and an incorrect response to a sample positive-feedback learning trial and an example of a correct and incorrect response to a sample negative-feedback learning trial. These examples used images other than those assigned to S1–S4. The actual task contained 160 trials, separated and randomized into four blocks. Trials were separated by a 2 s interval, during which time the screen was blank. Within each block, each stimulus appeared 10 times, 9 times with the more common outcome (e.g., category A for S1 and S3 and category B for S2 and S4), and once with the less common outcome. Thus, positive-feedback learning trials (S1 and S2) and negative-feedback learning trials (S3 and S4) were intermixed. On each trial, the computer recorded whether the participant made the optimal response (i.e., category A for S1 and S3 and category B for S2 and S4) regardless of the actual outcome.

### Mathematical Modeling

**Figure 2:**
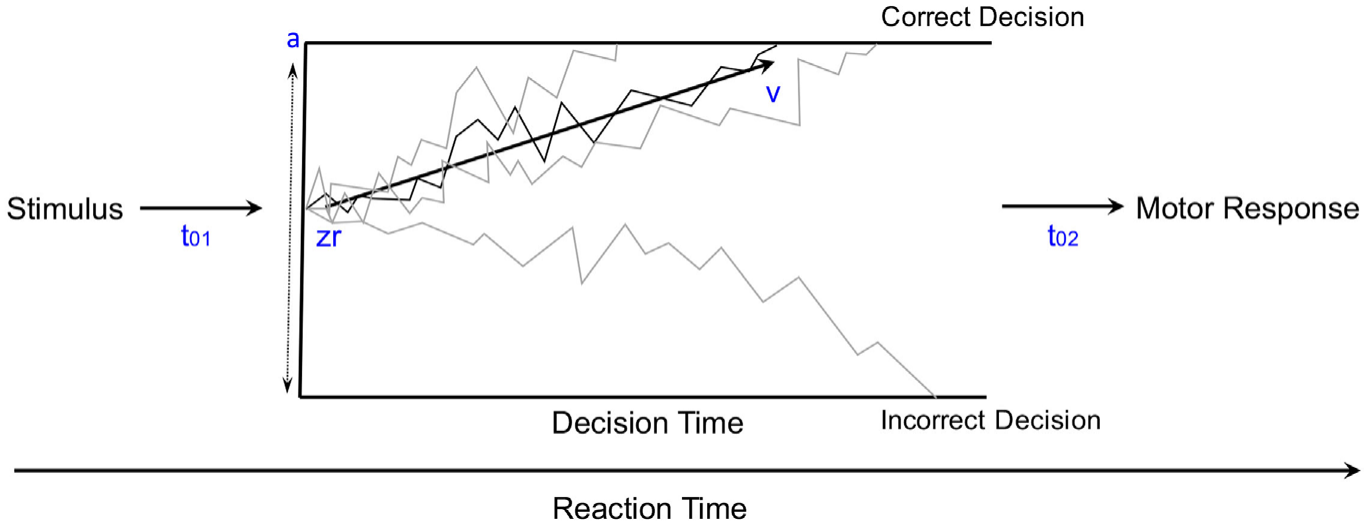
**The diffusion model of decision-making illustrated for pedagogical purposes with several different types of learning curves [modified from Ratcliff and McKoon (31)]**.

Mathematical modeling of the accumulation of evidence toward a decision threshold (the “drift model” of decision-making) offers a formal tool for differentiating between many key variables that underlie the learning process in probabilistic category-learning tasks such as that employed in the current study (31). This model encodes behavioral variables including accuracy, mean RT, and RT distributions into components of cognitive processing. After stimulus onset, a decision is reached via the accumulation of noisy evidence to a decision threshold (the correct decision being reached if the evidence reaches the top threshold). The functioning of this model is illustrated by the schematic in Figure 2 for several different types of learning curves (note: these are not the data from our study, but rather shown for pedagogical purposes only). The drift rate (*v*) describes the rate at which evidence is accumulated (examples shown by jagged lines in Figure 2) for the correct decision and can be thought of as the efficiency with which evidence is accumulated. The threshold separation (*a*) describes the amount of evidence required to make a decision, where a larger separation means greater caution in decision-making because more evidence must be accumulated before the decision threshold is reached. The relative starting point (zr) is a bias parameter, where the evidence accumulation process may be more or less biased toward the correct decision when zr is not equal to 0.5. Non-decision time (*t*0) accommodates for time to execute motor responses (*t*02, in this case a key press) and sometimes is also thought to include initial stimulus processing (*t*01), for example, from retina through early occipital cortex. Not shown explicitly on Figure 2 is the difference in decision time for correct and incorrect responses (*d*); where a positive *d* indicates faster responding when the correct response is chosen (as would be expected if incorrect decisions were made based on incredibly noisy evidence).

Five variants of a diffusion model were fit to the data from our current probabilistic category-learning studies. The basic diffusion model was implemented with five parameters: *a* (the threshold separation for making a decision), zr (the starting point of the evidence for making a decision—also known as a bias parameter), *v* (the drift rate or slope of the information accumulation process), *t*0 (non-decision time or time for response execution), and *d* (differences in speed of response execution for correct and incorrect responses), as shown in Figure 2. The five variations tested allowed each of these parameters to vary based on whether the stimulus was rewarding or punishing, in order to explain how differences in learning might arise at a cognitive level.

Models were fit using a maximum likelihood approach implemented in fast-dm 30 (40, 41). Data were first cleaned by removing trials where RT was shorter than 0.3 s or longer than 6 s [Ratcliff (42)—although the upper limit on the RT is usually 3 s, here this was raised to accommodate for the PD groups]. The model with the smallest negative log-likelihood was taken as the best fit (since all models contained the same number of parameters). The models are described by one of the five parameters that were varied, one at a time, based on whether the stimulus was rewarding or punishing while the other parameters were not set constant. The average fit is the overall average negative log-likelihood of the model, where smaller values indicate a better fit. The best fitting model was, therefore, the model where we varied the drift rate (*v*) based on whether the stimulus was rewarding or punishing. The fits are shown in Table 3.

**Table 3:** **Average fit of each model**.

Model	Average fit
*zr*	245.41
*a*	248.48
*v*	242.40
*t*0	247.74
*D*	248.14

### Statistical Analysis

The normality of sample distributions (e.g., subject’s accuracy and reaction times) was checked using Kolmogorov–Smirnov tests of the sample’s empirical distribution against a reference normal cumulative distribution function, with the null hypothesis that the sample is from a normal distribution. For the computer-based cognitive task, we used multivariate mixed-design ANOVA, two-way mixed-model ANOVA, one-way ANOVA, independent-samples *t*-test, and one-sample *t*-test. The level of significance was set at α = 0.05. Bonferroni corrections were used to protect significance level.

## Results

### Cognitive Task

We checked the normality of the data used in our analyses. All learning accuracy and RT data were normally distributed according to the Kolmogorov–Smirnov test (*p* > 0.1). Furthermore, we used one-sample *t*-test to assess whether subjects in different groups learned from positive and negative feedback better than chance (50%), with Bonferroni corrected α = 0.006 to protect significance level. We used the percentage of optimal responses in the fourth block of trials as the independent variable. Subjects in the HC and PD groups learned significantly better than chance from positive feedback but those in the PD-MDD and MDD groups did not [PD-MDD: *t*(12) = 1.104, *p* = 0.291; MDD: *t*(11) = 0.831, *p* = 0.424; PD: *t*(16) = 3.395, *p* = 0.004; and HC: *t*(14) = 7.962, *p* < 0.001]. For negative feedback, subjects in all groups learned significantly better than chance [PD-MDD: *t*(12) = 4.629, *p* = 0.001; MDD: *t*(11) = 3.617, *p* = 0.004; PD: *t*(16) = 7.500, *p* < 0.001; and HC: *t*(16) = 7.231, *p* < 0.001].

**Figure 3:**
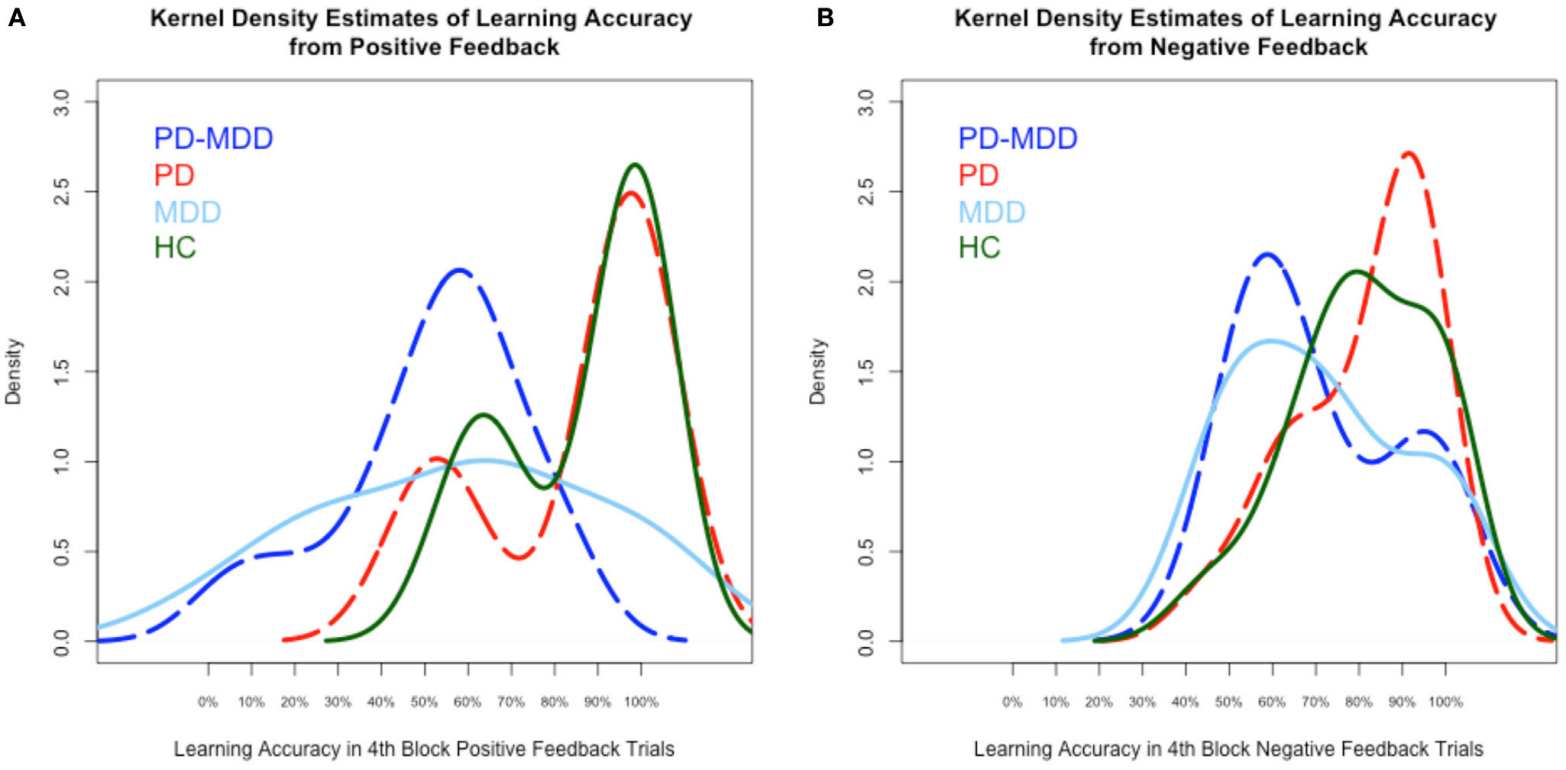
**Kernel density estimates of performance on the positive and negative feedback learning task**. **(A)** Density of subject scores in the fourth block of positive feedback stimuli. **(B)** Density of subject scores in the fourth block of negative feedback stimuli. In this and subsequent graphs, PD, Parkinson’s disease; MDD, major depressive disorder; PD-MDD, PD with comorbid MDD; HC, healthy control.

We used kernel density estimates to qualitatively examine subjects’ learning accuracy and RT in the fourth block of positive and negative feedback trials. As shown in Figure 3A, the MDD groups’ (PD-MDD and MDD) highest density in learning from positive feedback (around 60% optimal responses) was non-overlapping with that of the PD and HC groups, which were almost identical (around 95% optimal responses). In Figure 3B, although the density peaks in learning from negative feedback were not clearly separated as in positive feedback, the MDD groups (PD-MDD and MDD, around 60% optimal responses) were different from the PD (around 90% optimal responses) and HC (around 90% optimal responses) groups.

**Figure 4:**
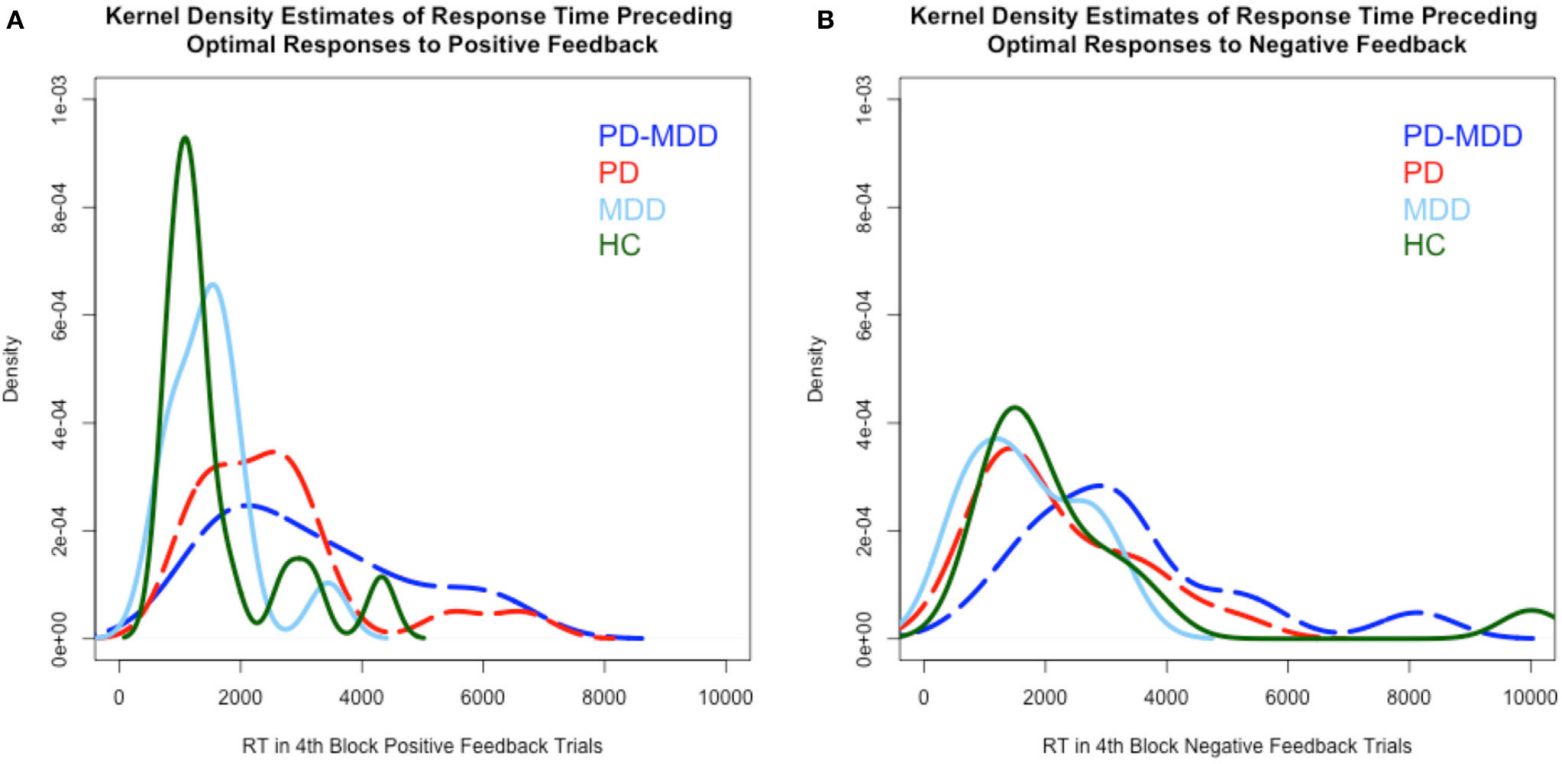
**Kernel density estimates of RT in positive and negative feedback trials. (A)** Density of subject RT in the fourth block of positive feedback stimuli. **(B)** Density of subject RT in the fourth block of negative feedback stimuli. In this and subsequent graphs, PD, Parkinson’s disease; MDD, major depressive disorder; PD-MDD, PD with comorbid MDD; HC, healthy control.

RT kernel density estimates exhibited a different pattern than learning accuracy, as shown in Figure 4. In the RT to positive feedback, the PD groups’ (PD-MDD and PD) density peaks (around 2,000 ms) were different from the MDD peak (around 1,500 ms) and the HC peak (around 1,000 ms) as shown in Figure 4A. In the RT to negative feedback, as shown in Figure 4B, the density peak of PD-MDD group (around 3,000 ms) was different from the rest of the group peaks (all around 1,500 ms).

We used a multivariate mixed-model ANOVA to analyze our results, with feedback type (positive feedback and negative feedback) as the within-subject variable, groups PD (with PD and without PD) and MDD (with MDD and without MDD) as between-subject variables, and the mean percentage optimal response across blocks (positive feedback accuracy and negative feedback accuracy) as well as the average RT across blocks (RT to positive feedback and RT to negative feedback) as the dependent variables. Box’s test of equality of covariance matrices was significant [Box’s M = 71.565, *F*(30, 6,029.494) = 2.034, *p* = 0.001]. Therefore, we used *F* and *p* values generated by Pillai’s Trace criterion. Levene’s tests of equality of error variance and Mauchly’s test of sphericity were not significant.

As described below, there was a significant multivariate effect of PD and MDD. Furthermore, there were approaching significance interactions between feedback type and PD, feedback type and MDD, and feedback type and PD and MDD (η^2^ represents effect size and β represents *post hoc* statistical power).

**Table:** 

Variable(s)	*F*	df between	df error	*p*	η^2^	β
MDD	11.251	2	51	0.000	0.306	0.989
PD	3.955	2	51	0.025	0.134	0.685
MDD*PD	0.737	2	51	0.484		
Feedback	0.693	2	51	0.505		
Feedback*MDD	2.777	2	51	0.072	0.098	0.523
Feedback*PD	2.921	2	51	0.063	0.103	0.546
Feedback*MDD*PD	2.831	2	51	0.068	0.100	0.532

Univariate between-subject tests are summarized in the table below. Results revealed a significant effect of MDD on the accuracy dependent variable but not on the RT-dependent variable. Conversely, PD had a significant effect on the RT-dependent variable but not the accuracy-dependent variable. However, there was no interaction between MDD and PD in either accuracy or RT (η^2^ represents effect size, β represents *post hoc* statistical power).

**Table:** 

Variable(s)	DV	*F*	df between	df error	*p*	η^2^	β
MDD	Accuracy	22.937	1	52	0.000	0.306	0.992
	RT	0.254	1	52	0.616		
PD	Accuracy	1.035	1	52	0.314		
	RT	7.457	1	52	0.009	0.125	0.160
MDD*PD	Accuracy	0.019	1	52	0.891		
	RT	1.442	1	52	0.235		

Univariate within-subject comparisons revealed a significant effect of interaction between feedback type and MDD on accuracy, a significant effect of interaction between feedback type and PD on RT, and an effect of the three-way interaction between feedback type, MDD, and PD on RT. Results are summarized below (η^2^ represents effect size, β represents *post hoc* statistical power).

**Table:** 

Variable(s)	DV	*F*	df between	df error	*p*	η^2^	β
Feedback	Accuracy	1.031	1	52	0.315		
	RT	0.535	1	52	0.468		
Feedback*MDD	Accuracy	5.488	1	52	0.023	0.095	0.518
	RT	0.471	1	52	0.495		
Feedback*PD	Accuracy	1.019	1	52	0.318		
	RT	4.370	1	52	0.041	0.078	0.420
Feedback*MDD*PD	Accuracy	0.417	1	52	0.521		
	RT	5.633	1	52	0.021	0.098	0.530

**Figure 5:**
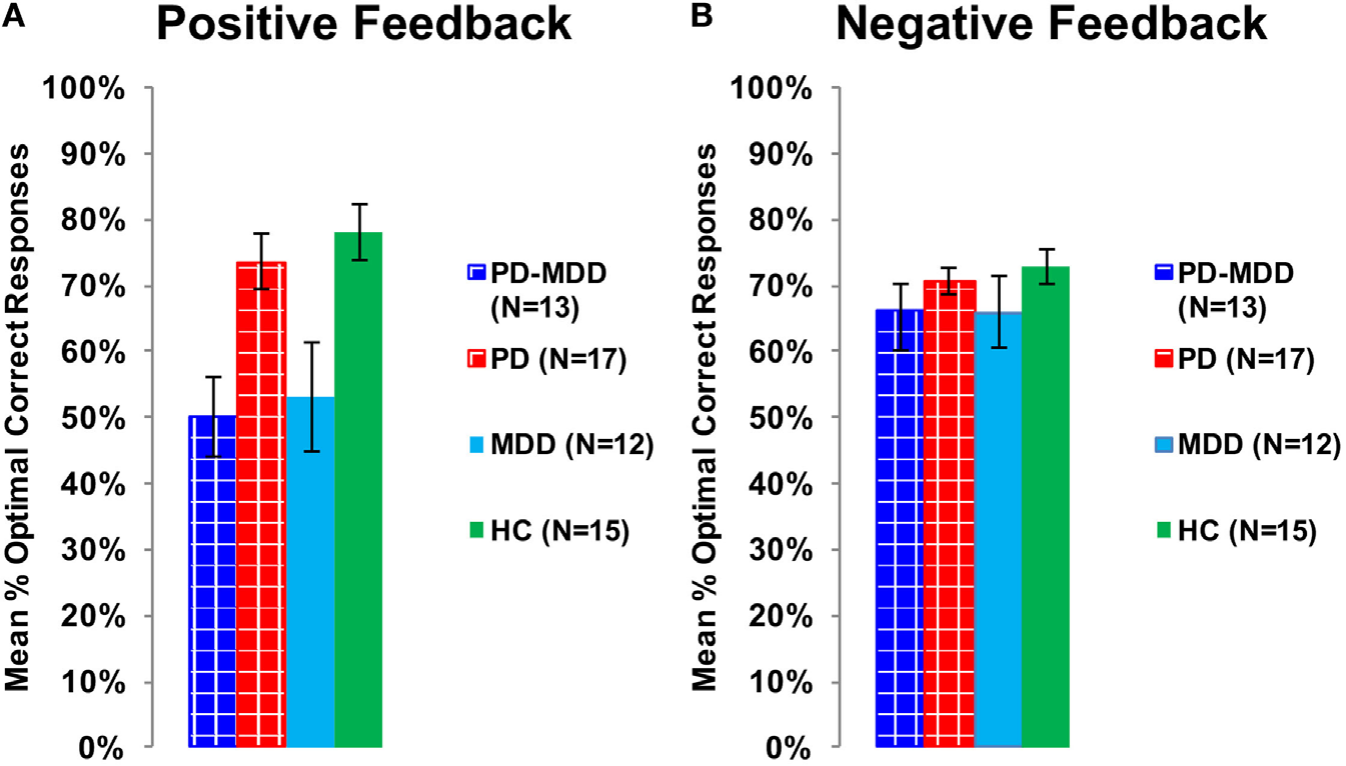
**Performance on the positive and negative feedback learning task**. **(A)** The mean percentage of optimal responses for the positive feedback stimuli. **(B)** The mean number of optimal responses in overall for the negative feedback stimuli. In this and subsequent graphs, PD, Parkinson’s disease; MDD, major depressive disorder; PD-MDD, PD with comorbid MDD; HC, healthy control; error bars show SEM.

To explore the effect of interaction between feedback type and MDD on accuracy, we used two independent-samples *t*-tests for accuracy feedback type (positive feedback and negative feedback), with the MDD group (with or without MDD) as the between-subject variable, and the mean percentage optimal response across blocks as the dependent variable (positive feedback accuracy and negative feedback accuracy). There was a significant effect of MDD on positive feedback accuracy [*t*(55) = −4.487, *p* < 0.001, Cohen’s *D* = 1.198; Figure 5A] but not on negative feedback accuracy [*t*(36.404) = −1.636, *p* = 0.133; Figure 5B].

**Figure 6:**
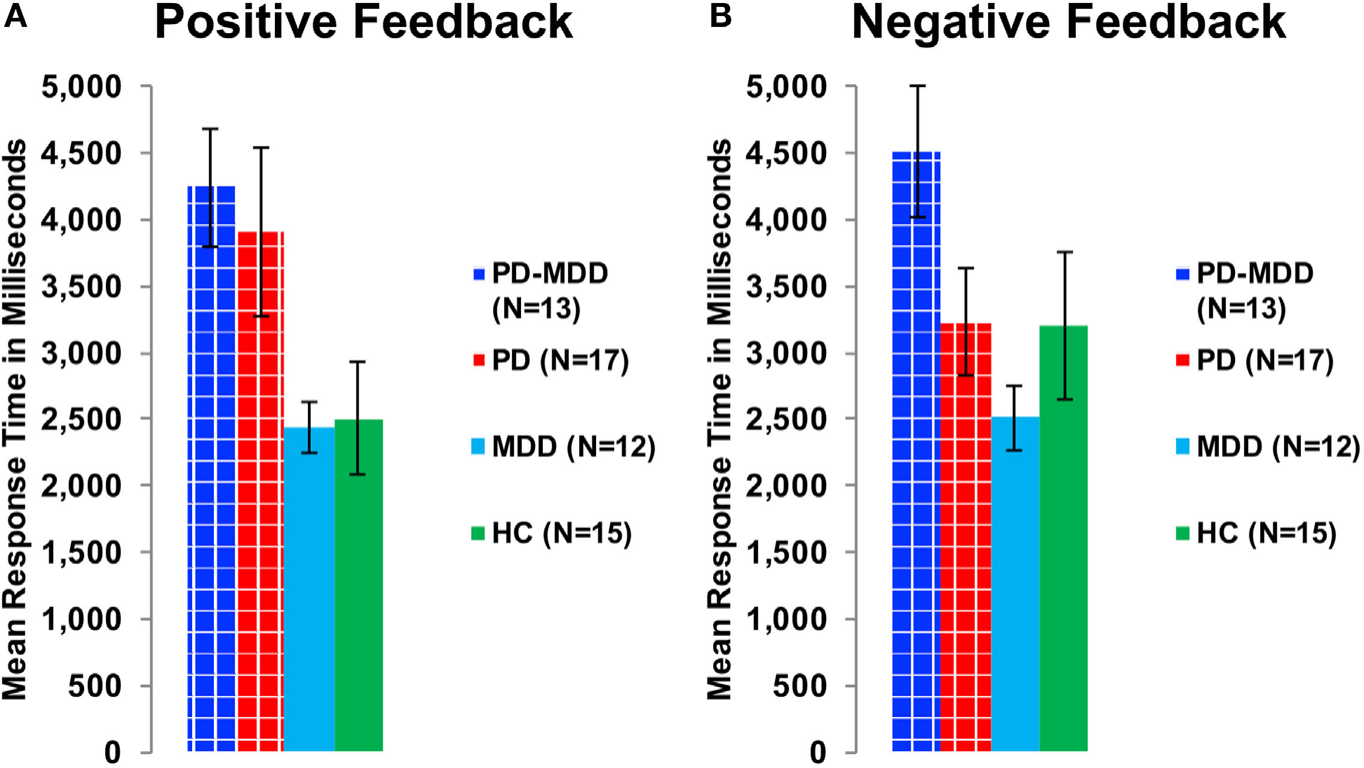
**RT in milliseconds during optimally answered positive and negative feedback learning trials**. **(A)** The mean RT during positive feedback trials. **(B)** The mean RT during negative feedback trials.

Furthermore, we used two independent-samples *t*-tests on RT to feedback types to study the interaction between feedback type and PD on RT. The PD group (with or without MDD) was the independent variable, while RT to positive or negative feedback was the dependent variable. There was a significant effect of PD on RT to positive feedback [*t*(48.041) = 3.172, *p* = 0.002, Cohen’s *D* = 0.850; Figure 6A] and an approaching-significance effect on RT to negative feedback [*t*(55) = 1.860, *p* = 0.068, Cohen’s *D* = 0.498; Figure 6B].

To investigate the effect of the three-way interaction between feedback type, MDD and PD on RT, we used two one-way ANOVA tests on RT to positive and negative feedback with group (PD-MDD, MDD, PD, and HC) as the between-subject variable. There was a significant effect of group on response in positive feedback [*F*(3, 53) = 3.322, *p* = 0.027, η^2^ = 0.161] and negative feedback [*F*(3, 53) = 2.843, *p* = 0.046, η^2^ = 0.139]. *Post hoc* Tukey’s *HSD* test on RT to positive feedback revealed no significant pairwise comparisons, although differences between PD-MDD, PD, MDD, and HC were approaching significance (<0.1). For RT to negative feedback, there was one *post hoc* significant difference between PD-MDD and MDD.

### Mathematical Modeling

To analyze modeling results similar to cognitive results, we also used two-way mixed-design ANOVA, with extracted parameter type as the within-subject variable [threshold separation (*a*), non-decision time (*s*), difference in decision time (*d*), relative starting point (zr), drift rate for positive feedback (*v*1), and drift rate for negative feedback (*v*2)], MDD (present or not) and PD (present or not) as between-subject variables, and values for the six parameters as the dependent variables. Box’s test of equality of covariance matrices was significant [Box’s M = 71.565, *F*(30, 6,029.494) = 2.034, *p* = 0.001]. Therefore, we used *F* and *p* values generated by the Pillai’s Trace criterion. Levene’s test of equality of error variance was not significant for all dependent variables except for *v*2 [*F*(3, 52) = 4.452, *p* = 0.007]. Mauchly’s test of sphericity also produced a significant result (Mauchly’s *W* = 0.381, χ^2^ = 48.334, df = 14, *p* < 0.001). Therefore, we reported the Greenhouse–Geisser-corrected df in all within-subject comparisons.

There was a significant effect of parameters [*F*(3.883, 201.908) = 341.341, *p* < 0.001, η^2^ = 0.868], a significant interaction between parameters and MDD [*F*(3.883, 201.908) = 3.939, *p* = 0.005, η^2^ = 0.070], and a significant interaction between parameters and PD [*F*(3.883, 201.908) = 2.550, *p* = 0.042, η^2^ = 0.047]. There was no significant interaction between MDD, PD, and parameters [*F*(3.883, 201.908) = 0.600, *p* = 0.658]. Furthermore, there was neither a significant effect of MDD [*F*(1, 52) = 2.171, *p* = 0.147], PD [*F*(1, 52) = 1.309, *p* = 0.258], nor a significant interaction between MDD and PD [*F*(1, 52) = 1.046, *p* = 0.311].

**Figure 7:**
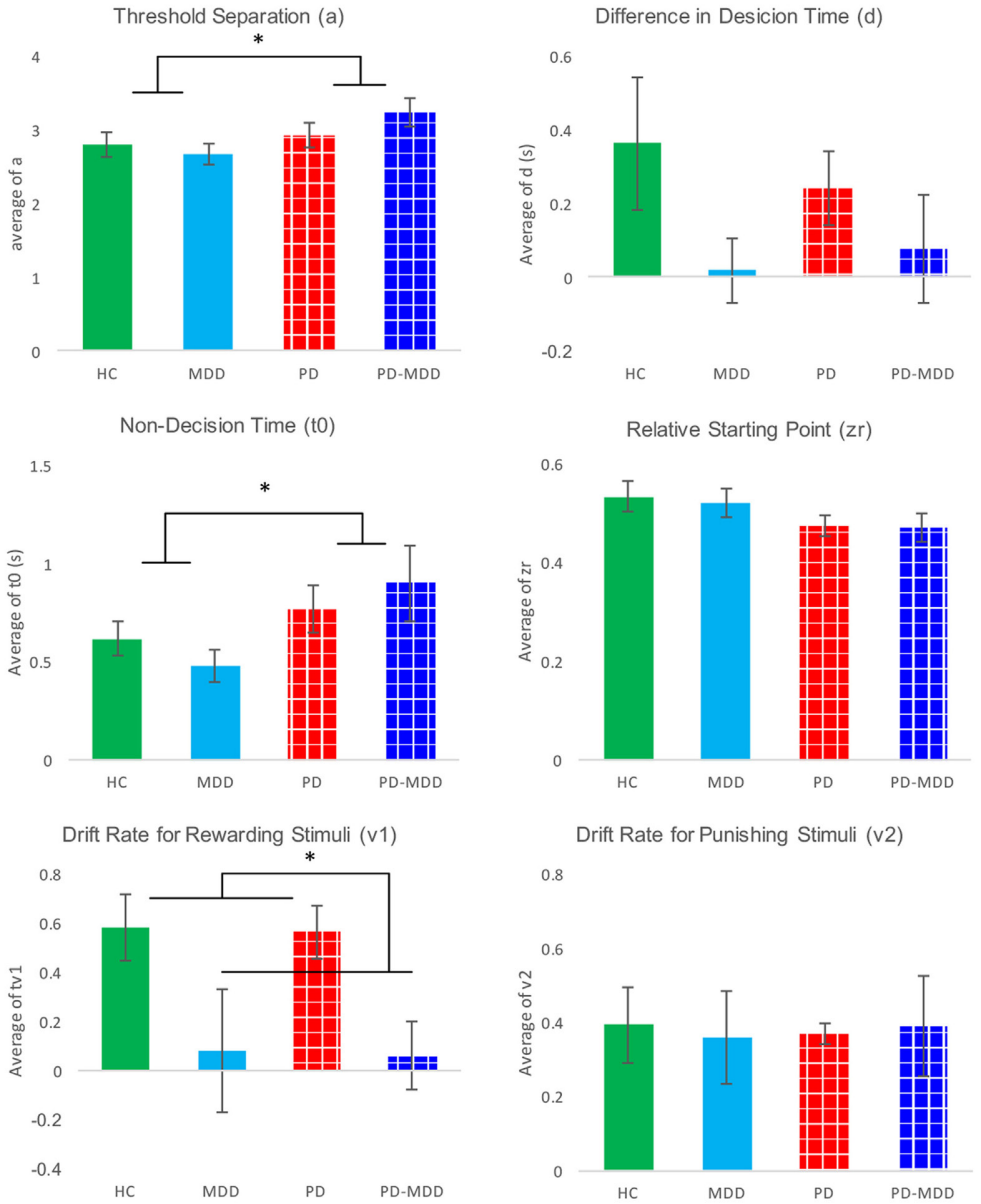
**Average parameter values for each group**. Significant differences are marked by *. These differences are shown between groups (participants with PD vs. not and participants with MDD vs. not).

We used six independent-samples *t*-tests to explore the interaction between parameters and MDD. The *t*-tests compared parameter values between the two MDD groups (PD-MDD and the MDD). There was a significant effect of MDD on the drift rate for positive feedback stimuli (Figure 7). Furthermore, there was an-approaching significance effect of MDD on the difference in decision time (Figure 7). Results are summarized below. *D* represents effect size as measured by Cohen’s *D*.

**Table:** 

Dependent variable	*t*	df	*p*	*D*
Threshold separation (*a*)	0.531	54	0.597	
Non-decision time (*s*)	0.498	54	0.989	
Difference in decision time (*d*)	−1.842	54	0.071	0.493
Relative starting point (zr)	−0.253	54	0.802	
Drift rate for positive feedback (*v*1)	−3.266	54	0.002	0.878
Drift rate for negative feedback (*v*2)	−0.53	36.694	0.960	

Furthermore, we investigated the interaction between parameters and PD using six independent-samples *t*-tests. The *t*-tests compared parameter values between the two PD groups (PD-MDD and the PD). As described below, PD had a significant effect on non-decision time and relative starting point. In addition, there was a short of significance effect of PD on threshold separation (Figure 7). *D* represents effect size as measured by Cohen’s *D*.

**Table:** 

Dependent variable	*t*	df	*p*	*D*
Threshold separation (*a*)	1.925	54	0.059	0.516
Non-decision time (*t*0)	2.219	45.424	0.031	0.594
Difference in decision time (*d*)	−0.244	54	0.808	
Relative starting point (zr)	−2.022	54	0.048	0.542
Drift rate for positive feedback (*v*1)	−0.023	54	0.981	
Drift rate for negative feedback (*v*2)	0.003	54	0.997	

## Discussion

The presence of MDD (in the PD-MDD and MDD groups) was associated with a selective deficit in learning accuracy from positive feedback. Conversely, the presence of PD (in the PD-MDD and PD groups) was associated with slower RT to positive feedback stimuli. Modeling results showed that the MDD groups had similar drift rates toward positive feedback and, therefore, learned less efficiently. On the other hand, the PD groups had higher threshold and, therefore, needed more evidence to make responses. To the best of our knowledge, this is the first study to examine learning from positive and negative feedback in PD with MDD, while properly controlling for the effects of PD and MDD.

It is well established that PD-MDD exacerbates cognitive decline associated with PD (13–16). In particular, our previous research suggests that patients with both PD and MDD were significantly more impaired on associative learning than patients with PD alone (43). Imaging studies found that higher BDI scores in patients with PD were associated with lower ability to utilize positive feedback (44). These results are in agreement with the current findings that patients with PD-MDD are impaired on learning from positive feedback, which also extend previous literature in an Arabic-speaking population.

Our results show that the choice (decision making) performance of patients with PD-MDD resembled that of patients with MDD. Converging evidence suggests that MDD, similar to unmedicated PD, is associated with a selective deficit in learning from positive feedback (19, 22, 23, 45, 46). In our study, medicated patients with PD learned as well as healthy subjects from positive feedback, while medicated PD-MDD and medication-naïve MDD patients did not. This argues that the positive feedback learning deficit in PD-MDD might be attributed to the MDD part of PD-MDD.

Dopaminergic neurotransmission has been associated with various cognitive domains (47). Previous studies found that patients with PD-MDD had more pronounced dopaminergic dysfunction in the substantia nigra pars compacta when compared to PD patients without MDD (48). Furthermore, both patients with PD and MDD showed reduced activation during reward anticipation in the putamen, caudate, nucleus accumbens, and dorsal anterior cingulate (49–51). Conversely, some studies suggested that striatal dopamine deficiency did not correlate with indices of MDD in PD, suggesting an extra striatal or non-dopaminergic mechanism (52, 53). In our study, although patients with PD-MDD were on dopaminergic medications, it does not seem that this remediation of dopamine levels affected cognitive function.

Patients with PD-MDD and patients with PD were significantly slower than MDD and HCs in responding to positive feedback stimuli. It is evident from our data that this deficit in RT is associated with the PD part of PD-MDD, not the MDD part. This could not be attributed to motor slowness in PD (25, 26) given that there was no difference between groups in response to negative feedback stimuli.

Relying on behavioral results only, it is not possible to tease apart the effects of processing speed, accumulation of cognitive evidence, rate of accumulation of cognitive evidence, and valence. Hence, we used a diffusion mathematical model to further analyze our behavioral data. Modeling showed that the RT differences for PD and PD-MDD groups were not simply the result of motor differences, but could also be influenced by patients with PD requiring more evidence to make their responses, as seen by the effect of PD on the estimate of their threshold separation (*a*) (Figure 7). Patients with PD (both PD and PD-MDD) showed significantly greater non-decision time (*t*0) as expected. However, their decision-making also differed in their threshold separation (*a*) revealing them to be more cautious responders, as they require more evidence to be collected before making a response. Patients with PD also tended to show a reduced bias; however, this did not reach significance. The difference in accuracy in learning from positive feedback seen in patients with MDD (MDD and PD-MDD groups) was explained by a significant difference in their drift rate for the positive feedback stimuli (*v*1). This difference was not present in the drift rate for negative feedback stimuli (*v*2) (Figure 7). This means that compared to HCs and patients with PD and not MDD, the MDD and PD-MDD groups were accumulating evidence for responses to positive feedback stimuli far slower, which could be the result of an impairment in learning from positive feedback. There was also a trend for a reduced *d* (difference in speed of response) in patients with MDD; however, this did not reach significance.

**Figure 8:**
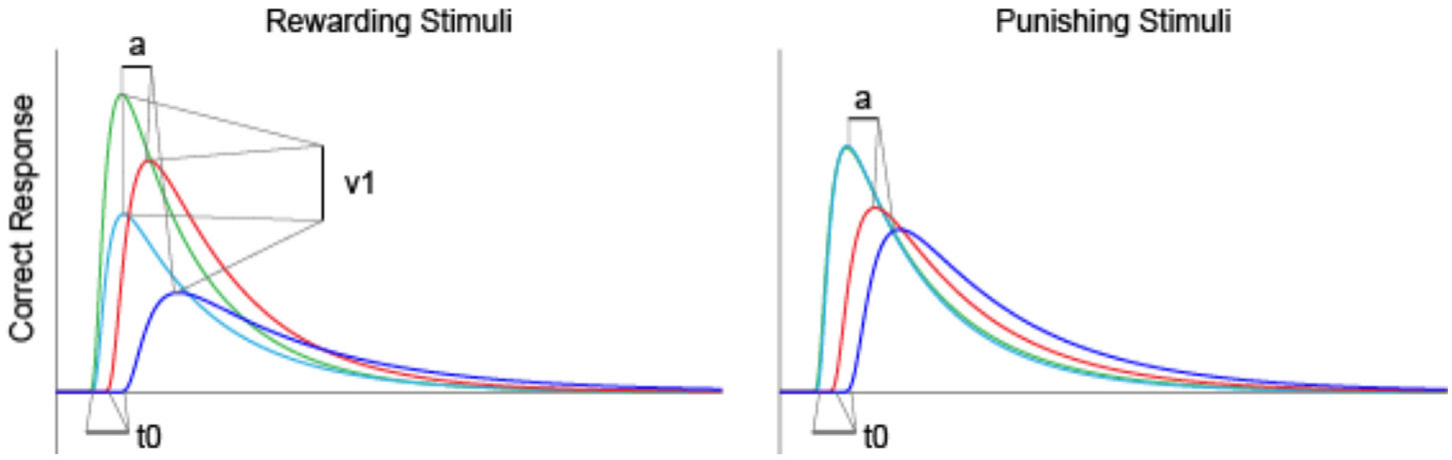
**Predicted RT distributions based on average parameter values for each group**. Only parameter values that were found to differ significantly between groups are outlined. Area under the curve represents density predicted RT of subjects.

As shown in Figure 8, we used average parameter values extracted from our model to calculate predicted RT density functions for the four groups. The peak positions in the density distribution match those in Figure 4 from experimental RT data. As predicted, non-decision time (*t*0) was found to be significantly longer in PD and PD-MDD groups, this can be seen from the longer time taken for the response distribution to begin reaching threshold (where the distribution “takes off” from the *x*-axis). Increased threshold separation (*a*) results in the distribution having a greater variance, as can be seen from the separation of the peaks along the *x*-axis, where the PD and PD-MDD groups show significantly greater threshold separation. The slope of the evidence accumulation for positive stimuli (*v*1) also differed between groups where patients with MDD and PD-MDD showed a slower evidence accumulation rate. This can be seen from the height of the peaks of the distributions, with a lower peak for correct responses and a larger peak for incorrect responses in MDD and PD-MDD groups.

### Limitations and Future Directions

All PD patients in our study were maintained on regular doses of dopaminergic medication. Furthermore, the MDD group received no medications. There was no way to examine whether the PD-associated deficit predated the MDD-associated deficit, or the opposite, in the PD-MDD group. Future studies ought to focus on the effects of dopaminergic medications on cognitive function in PD-MDD. Furthermore, MDD should be studied longitudinally in medication-naïve patients with PD to investigate the chronological order of cognitive deficits associated with PD-MDD.

## Ethics Statement

This study was carried out in accordance with the research protocol that was approved by the Al-Quds University Research Ethics Committee. Written informed consent was obtained from all subjects in accordance with the Declaration of Helsinki.

## Author Contributions

MH: study design, cognitive and modeling data analysis, and manuscript writing. HK: data collection, demographic data analysis, and manuscript writing. AT and AE: data collection, neuropsychological data analysis, and manuscript writing. HM: data collection and manuscript writing. MT: data collection and manuscript writing. ZG, MS, and AM: patient recruitment and clinical assessment. TB: computational modeling, modeling data analysis, and manuscript writing. AM: computational modeling and manuscript writing. CM and MG: manuscript writing.

## Conflict of Interest Statement

The authors declare that the research was conducted in the absence of any commercial or financial relationships that could be construed as a potential conflict of interest. The reviewer, DJ, and handling editor declared their shared affiliation, and the handling editor states that the process nevertheless met the standards of a fair and objective review.
